# How the Crosslinking Agent Influences the Thermal Stability of RTV Phenyl Silicone Rubber

**DOI:** 10.3390/ma12010088

**Published:** 2018-12-27

**Authors:** Chen He, Boqian Li, Ying Ren, Wu Lu, Yibing Zeng, Weidong He, Anchao Feng

**Affiliations:** 1 Aerospace Research Institute of Materials & Processing Technology, Beijing 100076, China; liboqian93@163.com (B.L.); xiaoyaojingily@163.com (Y.R.); 18330207345@163.com (W.L.); 15933573086@163.com (Y.Z.); 2 Key Laboratory of Soft Matter Chemistry, Chinese Academy of Sciences, and Department of Polymer Science and Engineering, University of Science and Technology of China, Anhui 230026, China; wdhe@ustc.edu.cn; 3 College of Materials Science and Engineering, Beijing University of Chemical Technology, Beijing 100029, China; fengac@mail.buct.edu.cn

**Keywords:** RTV phenyl silicone rubber, thermal stability, pyrolysis, cross-linking agent, residual hydroxyl

## Abstract

In this work, a thermal degradation mechanism of room temperature vulcanized (RTV) phenyl silicone rubber that was vulcanized by different crosslinking agents was discussed. Firstly, RTV phenyl silicone rubber samples were prepared by curing hydroxyl-terminated polymethyldiphenylsiloxane via three crosslinking agents, namely, tetraethoxysilane (TEOS), tetrapropoxysilane (TPOS), and polysilazane. Secondly, the ablation properties of RTV phenyl silicone rubber were studied by the muffle roaster test and FT-IR. Thirdly, thermal stability of the three samples was studied by thermogravimetric (TG) analysis. Finally, to explore the thermal degradation mechanism, the RTV phenyl silicone rubber vulcanized by different crosslinking agents were characterized by TG analysis-mass spectrum (TG-MS) and pyrolysis gas chromatogram-mass spectrum (pyGC-MS). Results showed that the thermal stability of RTV phenyl silicone rubber is related to the amount of residual Si–OH groups. The residual Si–OH groups initiated the polysiloxane chain degradation via an ‘unzipping’ mechanism.

## 1. Introduction

Silicone is the compound of Si atoms connected with organic groups through chemical bonds [1]. It is more thermally stable than other organic compounds, resulting from the many chemical bonds containing Si atoms that have a higher bond energy, such as the Si–O bond, the Si–C bond, or the Si–N bond. Room temperature vulcanized (RTV) silicone rubber is linear polysiloxane with a high molecular weight and can be vulcanized at room temperature. Among the silicone family, RTV silicone rubber is more flexible, easier to be vulcanized, and has a lower glass transition temperature than other silicone compounds. Hence, it has been extensively used as a polymer matrix of thermal protection systems (TPSs) in the field of aerospace, given its excellent thermal stability, favorable flexibility, and better low temperature resistance [2,3,4,5,6,7,8]. For example, the Martin company utilized silicone rubber as the polymer matrix of the TPS of the Mars probe (SLA-561) [4].

In the field of aerospace, TPS is one of the key technologies which can decide the success and failure of an aircraft launch. As the thermal stability and degradation process of the silicone rubber matrix significantly influences the thermal protection effect of silicone rubber-based TPSs, many experts have focused on the thermal degradation mechanism of methyl silicone rubber and have fully studied the simple chemical structure of this material [9,10,11,12,13,14]. Generally, methyl silicone rubber is degraded by three kinds of reaction mechanisms in the inert environment, that is, ‘unzipping’, ‘random scission’, and ‘external catalysis’ [9]. Camino et al. reported the ‘unzipping’ thermal degradation mechanism of methyl silicone rubber [11,12]. These researchers found that the hydroxyl groups at the polymer chain ends initiate the thermal degradation of methyl silicone rubber via a ‘back-bite’ reaction. Then, the polymer chain of methyl silicone rubber would degrade, and cyclic trimer and tetramer were produced. Hamdani et al. mentioned that methyl silicone rubber is degraded via the ‘random scission’ reaction [13]. These authors found that the thermal degradation reaction occurs randomly between the silicone backbone, and the ‘random scission’ reaction requires sufficient flexibility from the polymer chain segments, high polarity from siloxane bonds, and a higher thermodynamic stability in the degradation products than in the reagents. Grassie et al. indicated an ‘externally catalyzed’ thermal degradation mechanism; these researchers determined that KOH causes the accelerated decomposition of polymers by forming many short oligomers and siloxy ions [14].

Phenyl silicone rubber has several side groups of the polysiloxane backbone that are substituted by phenyl groups. Phenyl silicone rubber has a better thermal stability and a higher residue after ablation than methyl silicone rubber [15,16,17,18]. As the thermal degradation mechanism of phenyl silicone rubber is more complex than methyl silicone rubber in the inert atmosphere, the mechanism should be studied by utilizing test methods more exactly. Grassie et al. studied the pyrolysis products of vinyl-terminated polymethyldiphenylsiloxane and vinyl-terminated polymethylphenylsiloxane by pyrolysis gas chromatograph-mass spectrum (pyGC-MS); they found that methyl and methyl phenyl cyclic oligomers are produced during pyrolysis [15,16]. However, pyGC-MS could just show the pyrolysis product at one temperature point; it could not monitor the whole pyrolysis process of phenyl silicone rubber. Jovanovic and coworkers studied the pyrolysis process of phenyl silicone rubber by thermogravimetric (TG) analysis, but it could not give out the pyrolysis product and pyrolysis mechanism of phenyl silicone rubber [17]. Deshpande et al. studied the pyrolysis residues by ^29^Si-NMR and element analysis; this test method could not give out the chemical composition of the pyrolysis gas [18]. All of the reports did not give out the pyrolysis mechanism of phenyl silicone rubber during the whole pyrolysis process.

In the present work, the thermal degradation mechanism of RTV phenyl silicone rubbers vulcanized by different crosslinkers was studied systematically. By combining element analysis and FT-IR after muffle roaster ablation, the thermal stability of phenyl silicone rubber vulcanized by different crosslinkers was studied qualitatively. Then, by a combination of TG analysis-mass spectrum (TG-MS) and pyGC-MS, the pyrolysis process of phenyl silicone rubber vulcanized by different crosslinkers was studied in detail. This work could help engineers to select the crosslinking agents of silicone-based TPSs theoretically, and it could guide engineers and researchers designing new types of crosslinking agents for silicone-based TPSs with better thermal stability.

## 2. Materials and Methods

### 2.1. Materials

Hydroxyl terminated polymethyldiphenylsiloxane was purchased from the Shanghai Resin company (Shanghai, China); tetraethoxysilane (TEOS), tetrapropoxysilane (TPOS), and dibutyl tin dilaurate (DBTDL) were purchased from Sinopharm (Shanghai, China); polysilazane was purchased from the Institute of Chemistry, Chinese Academy of Sciences (Beijing, China). The molecular weight of the polysilazane was about 1000 Da, and the kinematic viscosity was about 2000–3000 mpa s.

### 2.2. Preparation of the RTV Phenyl Silicone Rubber

Specifically, 100 g polymethyldiphenylsiloxane, 7 g crosslinking agents, and 0.5 g DBTDL were added into a 250 ml flask. After stirring for 10 min, the mixture was poured into a 200 mm × 200 mm × 2mm mold. The curing reaction was carried out for 7 days at room temperature.

### 2.3. Characterizations

#### 2.3.1. Muffle Roaster Test

The muffle furnace ablation test (CINITE, Beijing, China) was conducted according to the process as follows: Preparation of the sample with the size of 200 mm × 200 mm × 2 mm, then put the sample into a ceramic crucible and ablate it in the muffle roaster for 5 min.

#### 2.3.2. FT-IR Analysis

The samples were filmed on the surface of KBr pellets. The FT-IR spectra were recorded with a Tensor 27 Spectrometer (Bruker Optics, Karlsruhe, Germany). The measurements were carried out in the region of 4000–400 cm^−1^.

#### 2.3.3. Thermogravimetric (TG) Analysis

Thermogravimetric analysis was conducted using a STA 449 which was purchased from Netzsch (Selb, Germany). A 3 mg–10 mg sample was added into the alumina crucible. The heating process was from room temperature to 900 °C under N_2_ atmosphere with a heating rate of 10 °C/min.

#### 2.3.4. Thermogravimetric-Mass Spectrum (TG-MS)

TG-MS was conducted using a Thermo Mass Photo which was purchased from Rigaku (Tokyo, Japan). A 3 mg–10 mg sample was added into the alumina crucible. The heating process was from room temperature to 900 °C under N_2_ atmosphere with the heating rate of 10 °C/min.

#### 2.3.5. Pyrolysis Gas Chromatogram-Mass Spectrum (pyGC-MS)

A VG70-SE Sector mass spectrometer (VG, Manchester, Britain) with a gas chromatograph on the front end was employed for analysis of the volatile pyrolysis compounds collected. A DB-5 capillary column of 30 meters was employed for the gas chromatography column. At the end of each half-hour period, the glass ports of the trap were sealed. The liquid sample was warmed and a 10 cm^3^ sample of the gas space inside the trap was collected and analyzed via GC-MS. The mass fraction of evolved gases was obtained from areas under the peaks.

#### 2.3.6. Element Analysis

Element analysis was performed using an Energy Dispersive Spectrometer (Bruker, Karlsruhe, Germany) to characterize the distribution and content of elements. This also assisted with the analysis of the pyrolysis mechanism of RTV phenyl silicone rubber.

## 3. Results and Discussion

### 3.1. Curing Mechanism of RTV Phenyl Silicone Rubber with Different Crosslinking Agents

**Scheme 1:**
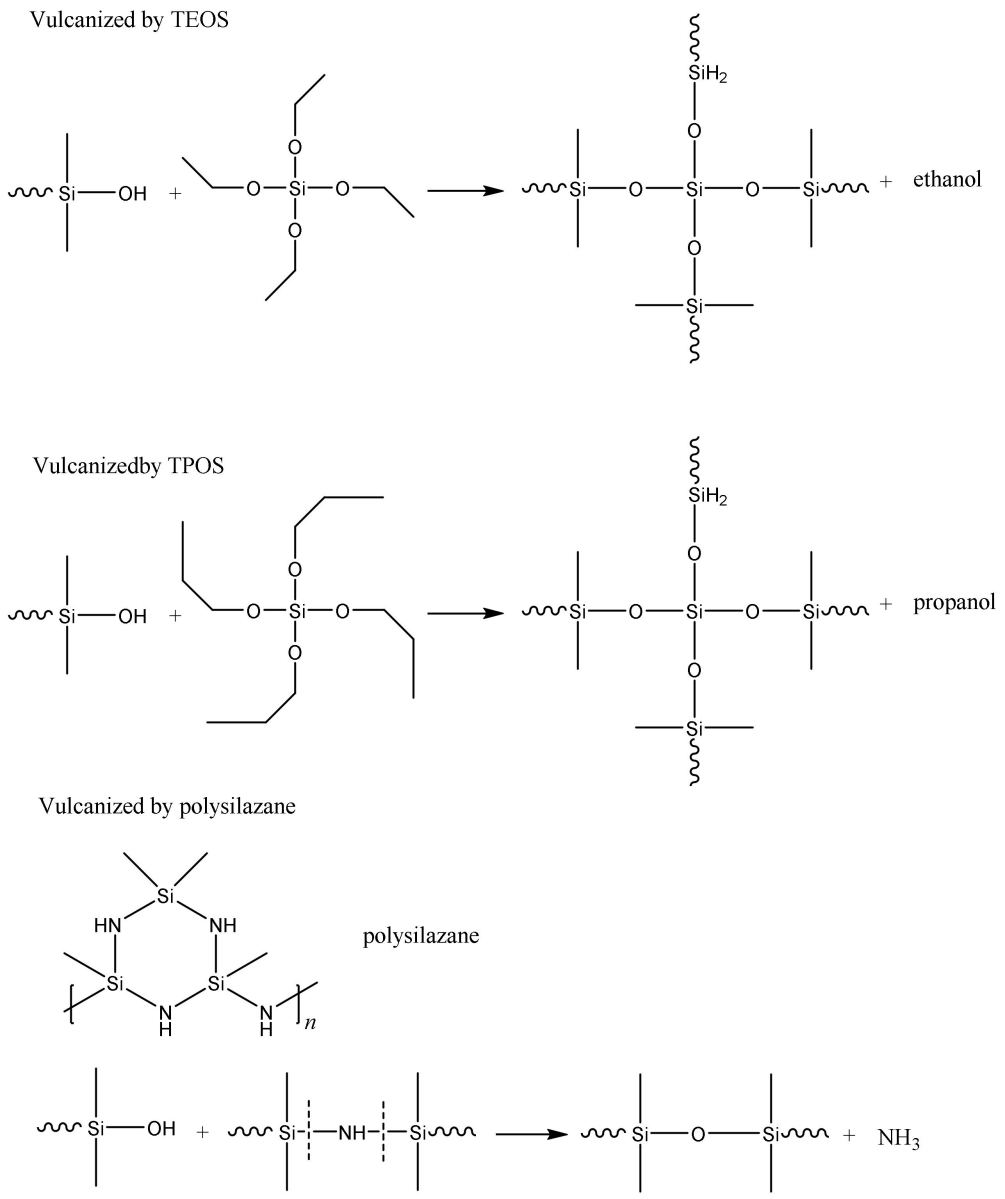
Vulcanized mechanisms of the phenyl silicone rubber. TEOS: tetraethoxysilane; TPOS: tetrapropoxysilane.

The curing mechanism of RTV phenyl silicone rubber with different crosslinking agents is shown in Scheme 1. For TEOS and TPOS, the curing mechanism was that the Si–OH condensed with C_2_H_5_O–Si or C_3_H_6_O–Si, and produced ethanol or propanol residues. For polysilazane, the Si–N bonds cleaved during the curing process, the Si–O–Si bonds were produced, and ammonia was given off.

### 3.2. Ablation Properties of RTV Phenyl Silicone Rubber

**Figure 1:**
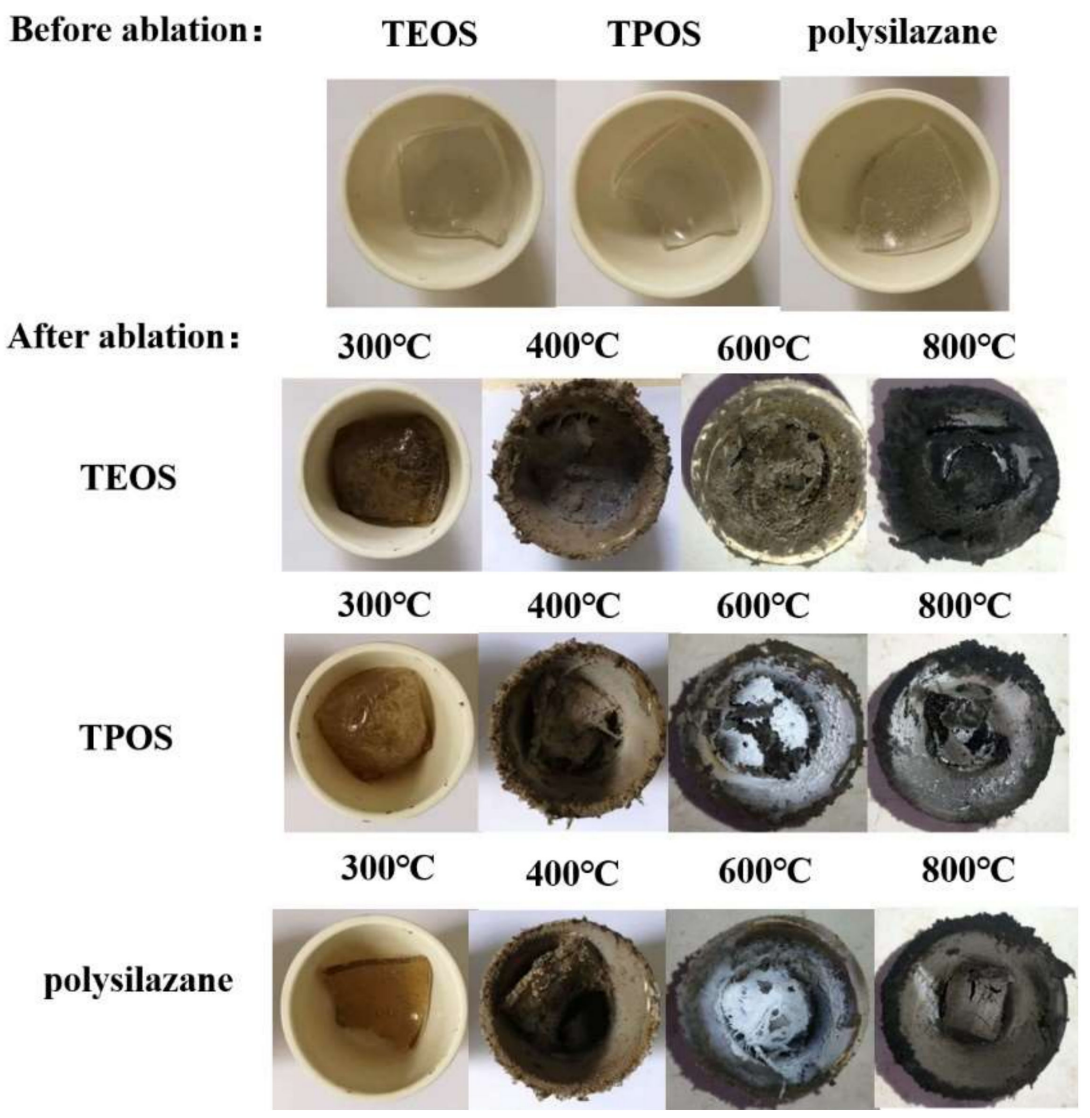
Muffle roaster test results of room temperature vulcanized (RTV) phenyl silicone rubber.

The results of the muffle roaster ablation test are shown in Figure 1. After ablation at 300 °C for 5 min, both the RTV phenyl silicone rubber vulcanized by TEOS and TPOS were softened, and a super bubble could be observed. However, the RTV phenyl silicone rubber vulcanized by polysilazane was still compact and hard. After ablation above 400 °C for 5 min, the RTV phenyl silicone rubber vulcanized by TEOS was ablated to ash. RTV phenyl silicone rubber vulcanized by TPOS could keep its shape after ablation at 400 °C for 5min, but it would ablate to ash when the ablation temperature was above 600 °C. For RTV phenyl silicone rubber vulcanized by polysilazane, it could keep its shape until the ablation temperature reached 800 °C, which means that RTV phenyl silicone rubber has excellent thermal stability.

Element analysis results of RTV phenyl silicone rubber after ablation are shown in Table 1. The percentage of C was decreased, and the percentage of Si and O were increased with the increasing of the ablation temperature, resulting from the fact that some of the C element was oxidized at high temperature. Besides, the percentage of C in RTV phenyl silicone rubber vulcanized by polysilazane decreased much slower than RTV phenyl silicone rubber vulcanized by TEOS and TPOS, which means that RTV phenyl silicone rubber vulcanized by polysilazane was more stable at a high temperature than RTV phenyl silicone rubber vulcanized by TEOS and TPOS.

**Figure 2:**
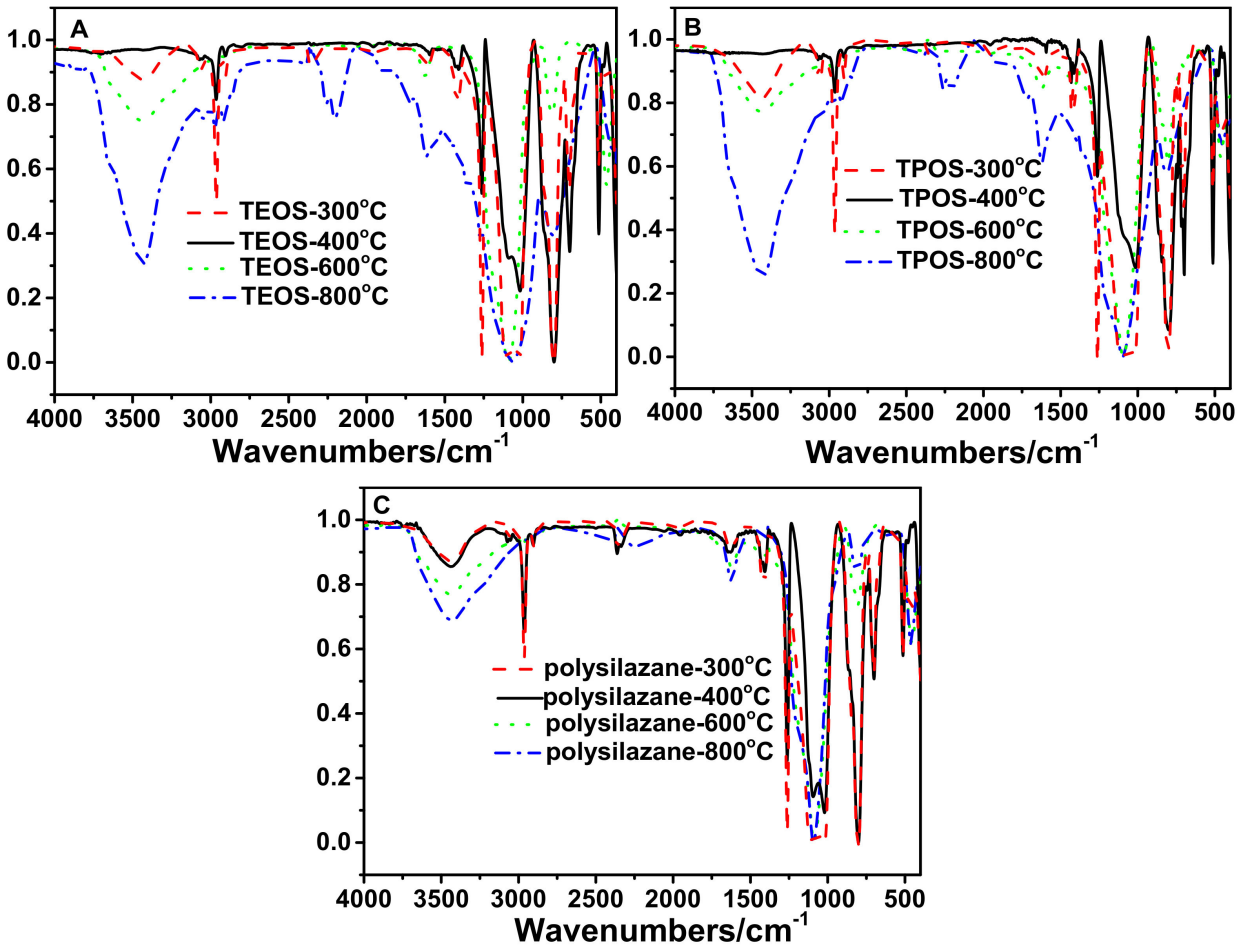
FT-IR spectra of the RTV phenyl silicone rubber residue after the muffle roaster test. (**A**) TEOS, (**B**) TPOS, (**C**) polysilazane.

FT-IR spectra of the RTV phenyl silicone rubber residue after the muffle roaster test are shown in Figure 2. The signals at 3400, 2934, 1261, and 1090 cm^−1^ are attributed to the stretching vibration of O–H, C–H, Si–CH_3_, and Si–O. As the ablated temperature increased, the signals of C–H and Si–CH_3_ decreased, and the signals of O–H and Si–O increased, resulting from the polysiloxane chain pyrolysis and amorphous SiO_2_ and amorphous Si–O–C ceramics produced. Besides, for RTV phenyl silicone rubber vulcanized by TEOS and TPOS, the two signals of C–H and Si–CH_3_ decreased sharply when they were ablated at 400 °C for 5 min. Whereas the two signals of RTV phenyl silicone rubber vulcanized by polysilazane were not reduced obviously, which demonstrated that RTV phenyl silicone rubber vulcanized by polysilazane has a better thermal stability at 400 °C.

### 3.3. TG Analysis

**Figure 3:**
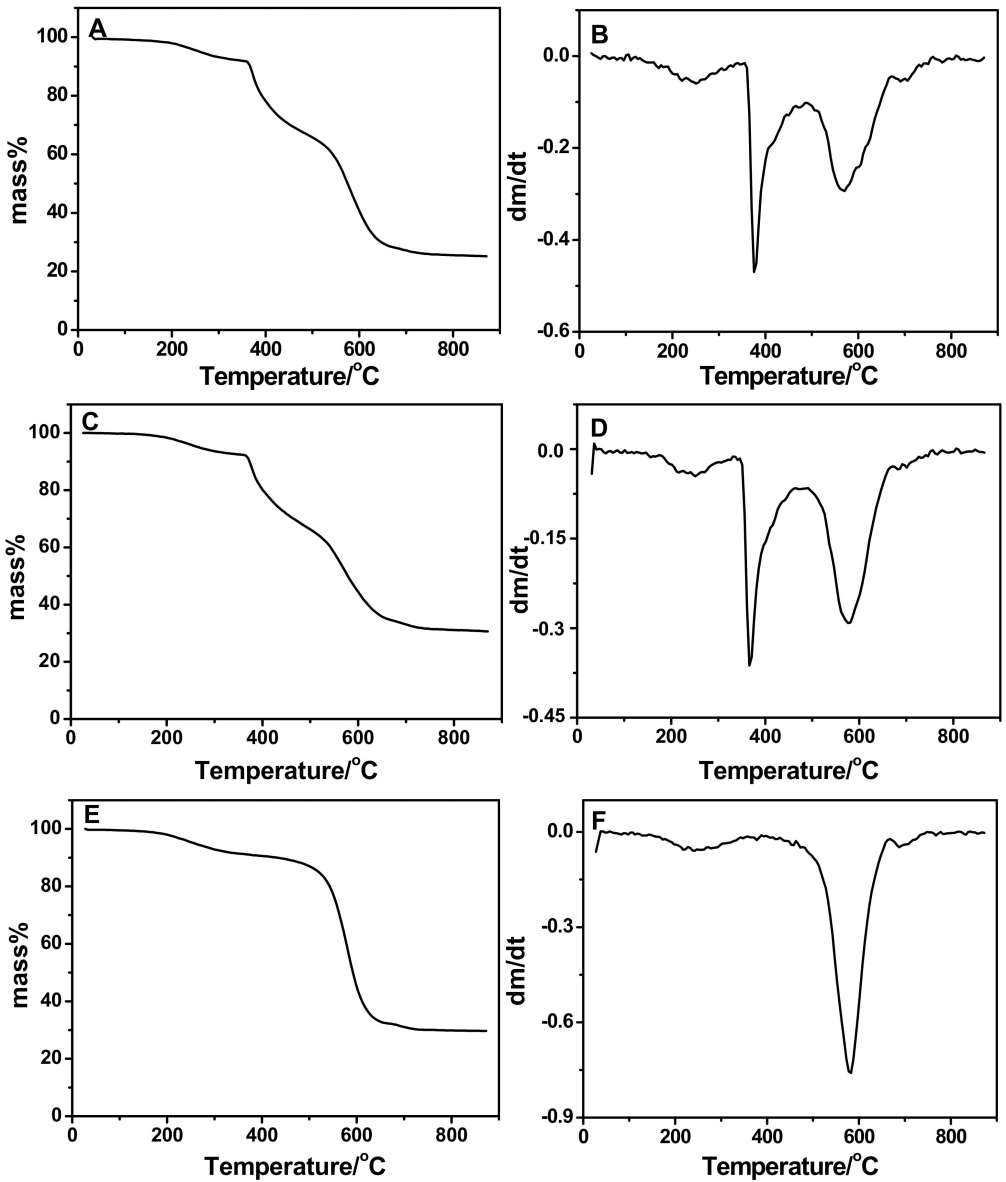
Thermogravimetric (TG) and Differential thermogravimetric (DTG) curves of RTV phenyl silicone rubber vulcanized by TEOS (**A** and **B**), TPOS (**C** and **D**), and polysilazane (**E** and **F**).

The TG analysis of the RTV phenyl silicone rubber vulcanized by different crosslinking agents is illustrated in Figure 3. The TG curve indicates that the thermal pyrolysis process of the RTV phenyl silicone rubber vulcanized by TEOS was divided into three stages. The temperature range of the first stage was from 190 to 320 °C, the second stage was from 320 to 480 °C, and the last stage was from 480 to 630 °C. From the TG curves, the final residue weight of the RTV phenyl silicone rubber was approximately 26%. The DTG (Differential thermogravimetric) curve of the thermal pyrolysis process also denotes that this method was divided into three steps, and the rate of the thermal degradation reaction had reached the maximum at approximately 380 °C. The thermal pyrolysis process of the RTV phenyl silicone rubber vulcanized by TPOS was similar to that of the RTV phenyl silicone rubber vulcanized by TEOS, as depicted in Figure 3C,D. This process could also be divided into three stages. However, the highest thermal degradation rate of the RTV phenyl silicone rubber vulcanized by TPOS was approximately 0.36% per minute, which was slower than the thermal degradation rate of the RTV phenyl silicone rubber vulcanized by TEOS. In contrast to the RTV phenyl silicone rubber vulcanized by TEOS and TPOS, the thermal degradation process of the RTV phenyl silicone rubber vulcanized by polysilazane was only divided into two stages. The temperature range of the first stage was from 190 to 320 °C and that of the final stage was from 480 to 630 °C; moreover, the thermal degradation rate reached the maximum at 580 °C. The residue rate of the RTV phenyl silicone rubber vulcanized by polysilazane was also 26%. The TG analysis showed that the thermal degradation rate of RTV phenyl silicone rubber was slower when vulcanized by polysilazane than by TPOS and TEOS.

### 3.4. TG-MS Analysis of RTV Phenyl Silicone Rubber

**Figure 4:**
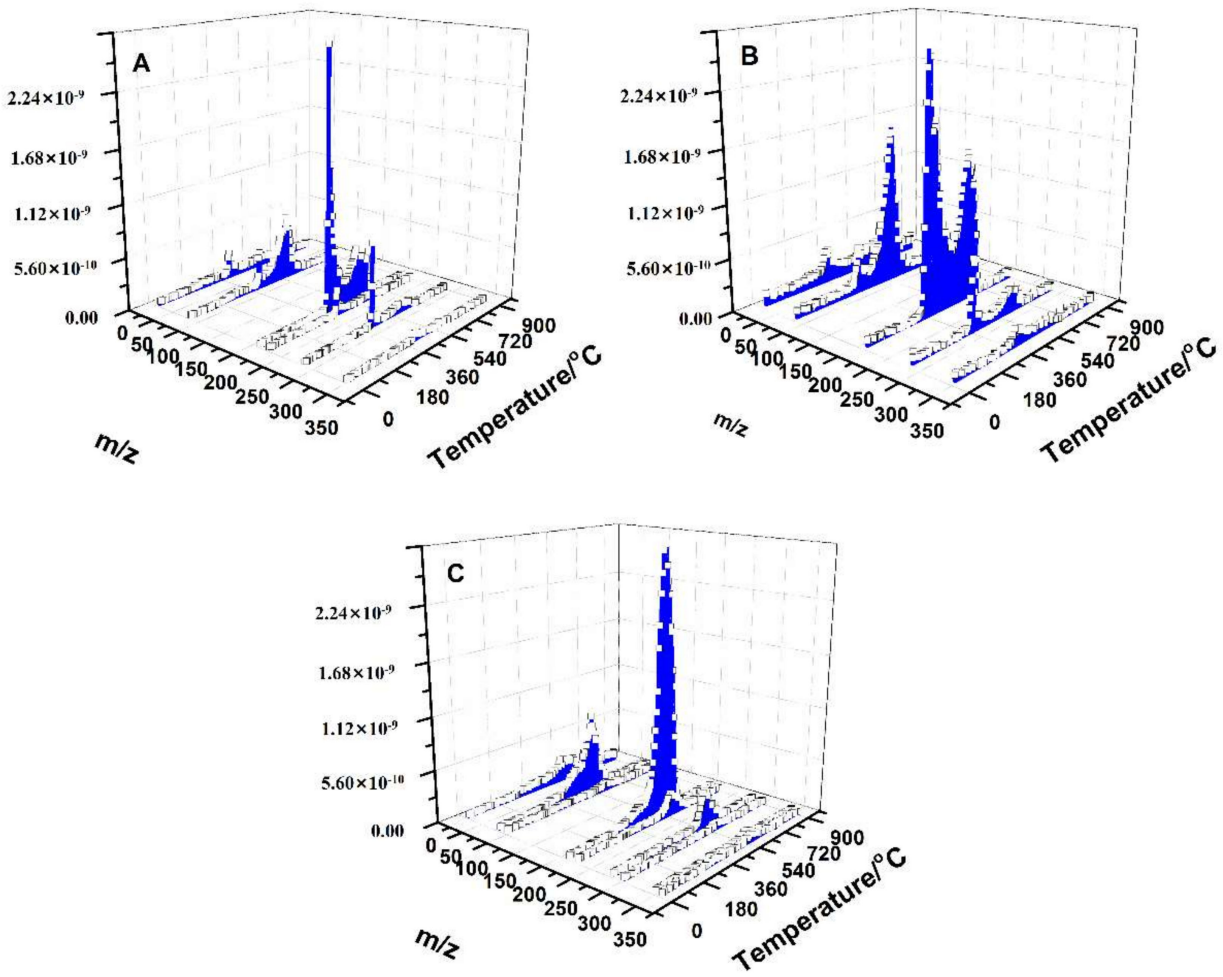
TG analysis–mass spectrum (TG-MS) results of RTV phenyl silicone rubber vulcanized by TEOS (**A**), TPOS (**B**) and polysilazane (**C**).

Furthermore, to explore the thermal pyrolysis product of RTV phenyl silicone rubber, TG-MS was used to characterize the product during the thermal pyrolysis reaction. Figure 4A exhibits the TG-MS results of the RTV phenyl silicone rubber vulcanized by TEOS. At the first thermal pyrolysis reaction stage (190–320 °C), the mass loss of the RTV phenyl silicone rubber was approximately 10%, but all signals were weak because of the cyclosiloxane impurity of the RTV phenyl silicone rubber vapor at this stage.

At the second thermal pyrolysis reaction stage (320–480 °C), the signal at the molecular weights of 222, 296, and 346 could be observed, thereby indicating that the RTV phenyl silicone rubber polymer chains were pyrolysed, and the pyrolysis residues were hexamethylcyclotrisiloxane, octamethylcyclotetrasiloxane, and diphenyltetramethylcyclotrisiloxane. In addition, the signal appeared at the molecular weights of 16 and 78, thus implying that a side group crosslinking reaction occurred and led to benzene and methane gas generation.

At the final thermal pyrolysis reaction stage (480–630 °C), the signal intensity declined at the molecular weights of 222, 296, and 346, but the signal intensity was enhanced at the molecular weights of 16 and 78, thereby denoting that the side group crosslinking reaction was enhanced at this stage.

The TG-MS results of the RTV phenyl silicone rubber vulcanized by TPOS were similar to those of the RTV phenyl silicone rubber vulcanized by TEOS, as shown in Figure 4B. By contrast, for the second thermal pyrolysis reaction stage, the intensity was weaker at the molecular weight of 222 than at TEOS, thus indicating that the polymer chain of the RTV phenyl silicone rubber pyrolysed less when vulcanized by TPOS than by TEOS at this stage. The results were similar to the apparent activation energy calculation results of the thermal pyrolysis reaction.

**Figure 5:**
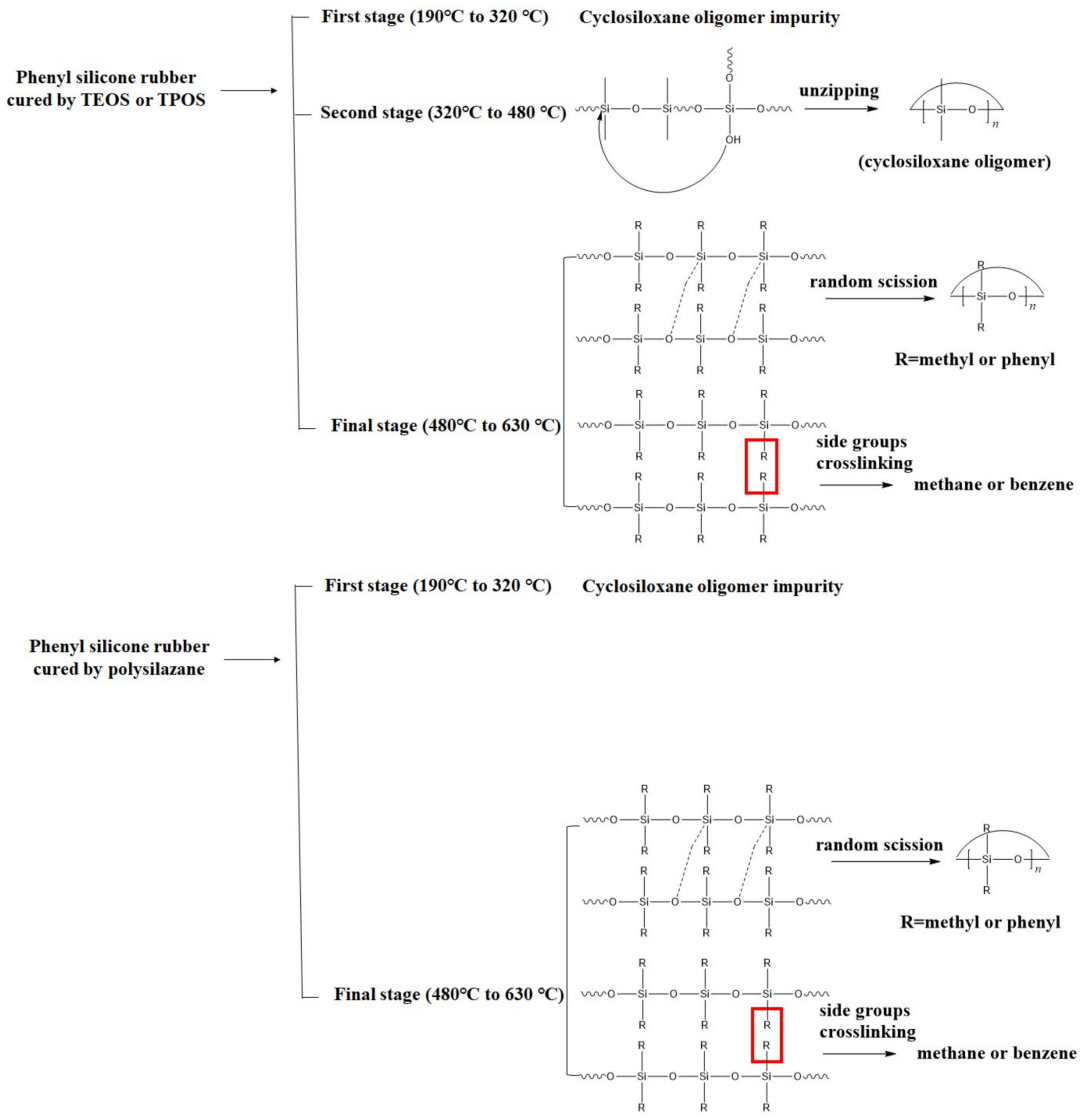
Summary of the pyrolysis reaction mechanisms of RTV phenyl silicone rubber vulcanized by TEOS, TPOS, and polysilazane.

The TG-MS results of the RTV phenyl silicone rubber vulcanized by polysilazane are displayed in Figure 4C. The curing mechanism of polysilazane presented in Figure 5 indicates that the hydroxyl groups would not produce residues and the ‘unzipping’ pyrolysis reaction did not occur. Thus, the RTV phenyl silicone rubber had a better thermal stability when vulcanized by polysilazane than by TEOS and TPOS given the absence of the ‘unzipping’ degradation process. The pyrolysis reaction was only divided into two stages and was caused by the cyclosiloxane oligomer impurity. The final pyrolysis reaction stage (480–630 °C) showed a signal intensity at the molecular weights of 222, 296, and 346, thereby indicating that the main polymer chain of the RTV phenyl silicone rubber vulcanized by polysilazane produced degraded and cyclosiloxane residues. Furthermore, the TG-MS results showed that the signal intensity was enhanced at the molecular weights of 16 and 78, thus denoting that the side group crosslinking reaction also occurred at this stage. Given the absence of hydroxyl residue, the main polymer chain depolymerisation was mainly initiated through ‘random scission’. Notably, the TG-MS signal intensity of the cyclosiloxane oligomers of the RTV phenyl silicone rubber was stronger when vulcanized by polysilazane than by TEOS or TPOS, and the apparent activation energy of the RTV phenyl silicone rubber was lower when vulcanized by polysilazane than by TEOS or TPOS.

### 3.5. pyGC-MS Analysis

**Figure 6:**
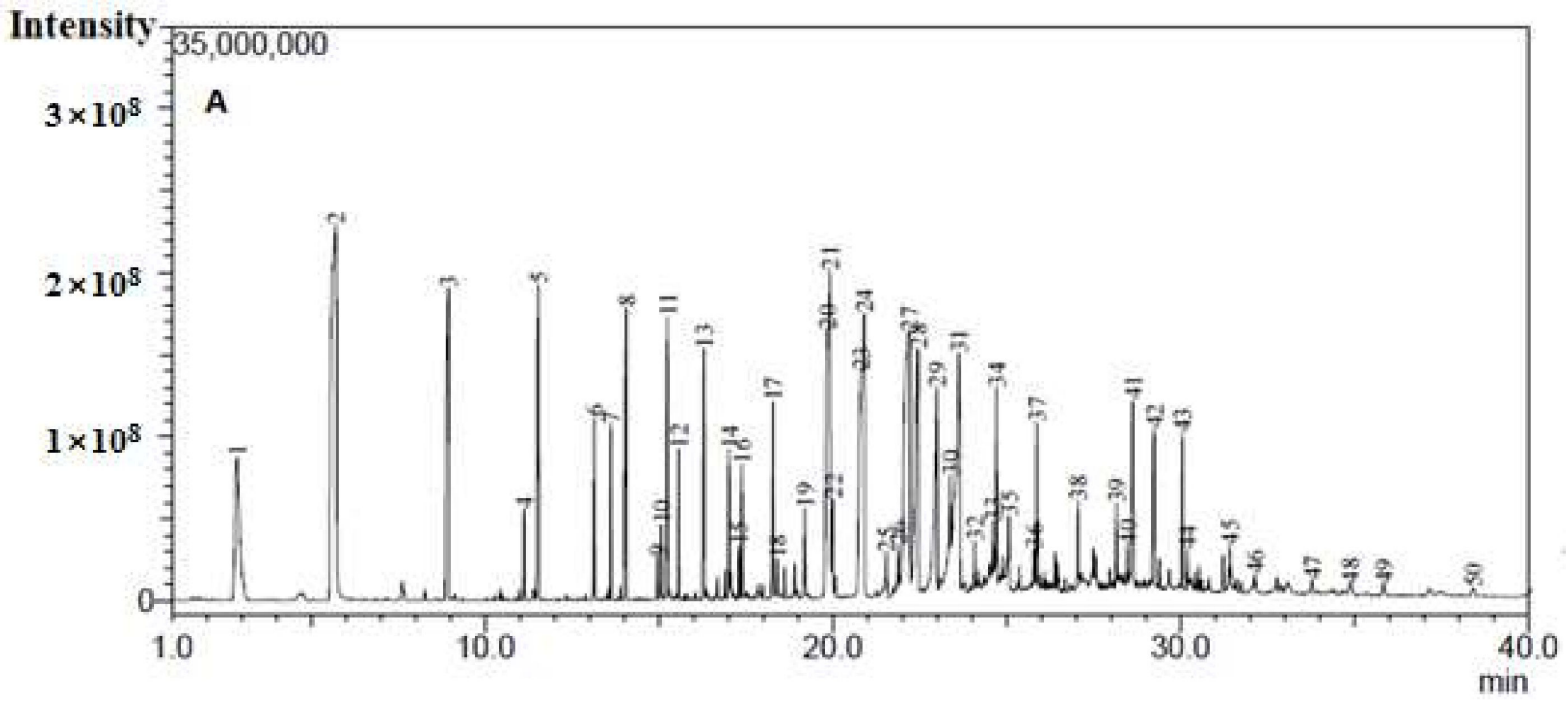

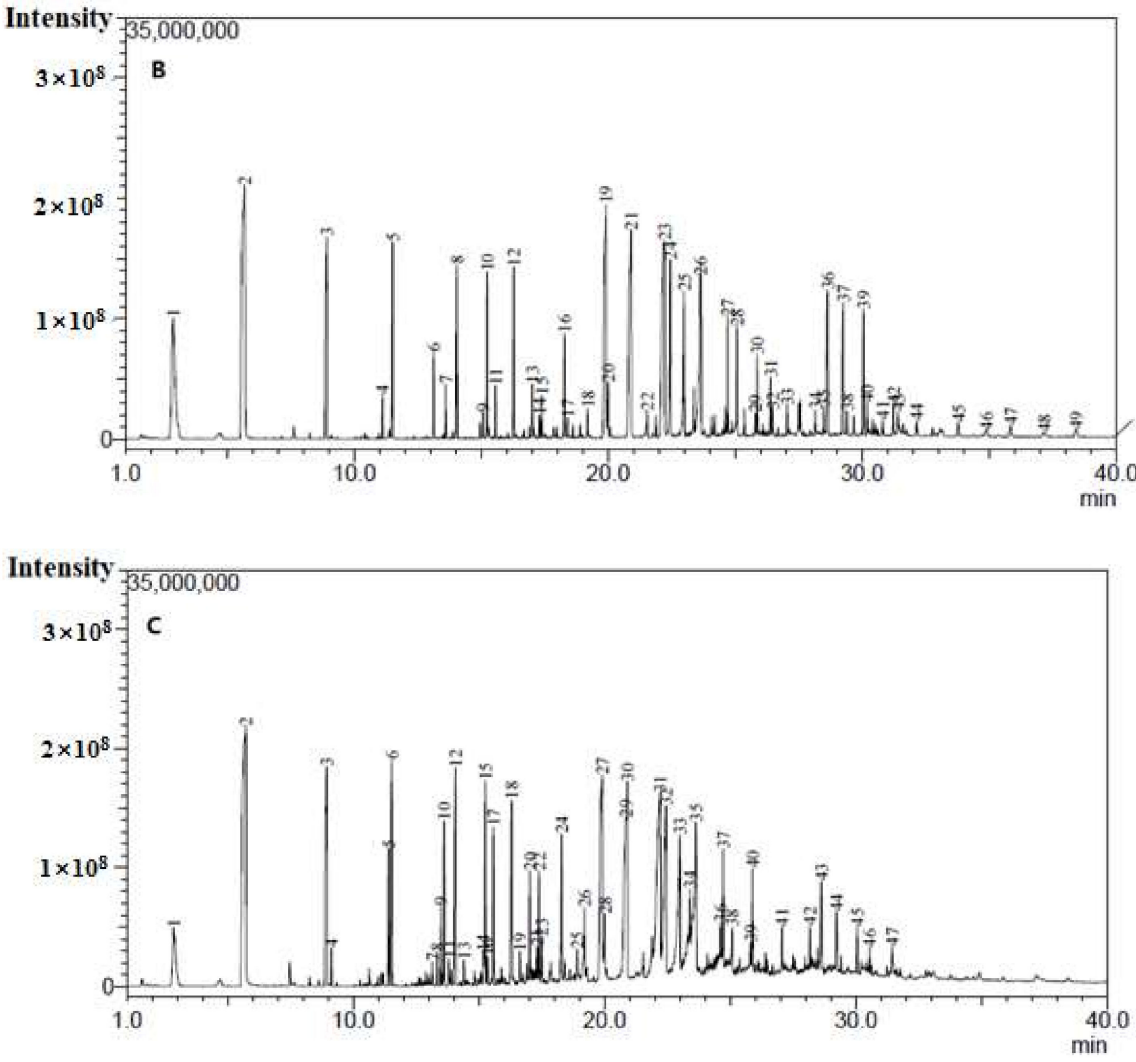
Pyrolysis gas chromatogram-mass spectrum (pyGC-MS) results of RTV phenyl silicone rubber vulcanized by TEOS (**A**), TPOS (**B**), and polysilazane (**C**).

PyGC-MS results are shown in Figure 6. The pyrolysis temperature was about 700 °C, which was a little higher than the final pyrolysis stage. Figure 6A is the pyGC-MS result of RTV phenyl silicone rubber vulcanized by TEOS. Peak 1 is methane, peak 2 is benzene, peak 3 to 50 is cyclosiloxane with different molecular weights and methyl or phenyl side groups. The pyGC-MS result of RTV phenyl silicone rubber vulcanized by TPOS and polysilazane are similar to RTV phenyl silicone rubber vulcanized by TEOS, as shown in Figure 6B,C. Peak 1 is methane, peak 2 is benzene, and other peaks are attributed to cyclosiloxane with different molecular weights and methyl or phenyl side groups. These results also confirmed that cyclosiloxane would be produced during the pyrolysis reaction.

### 3.6. Thermal Prolysis Mechanism of RTV Phenyl Silicone Rubber Vulcanized by Different Crosslinking Agents

**Figure 7:**
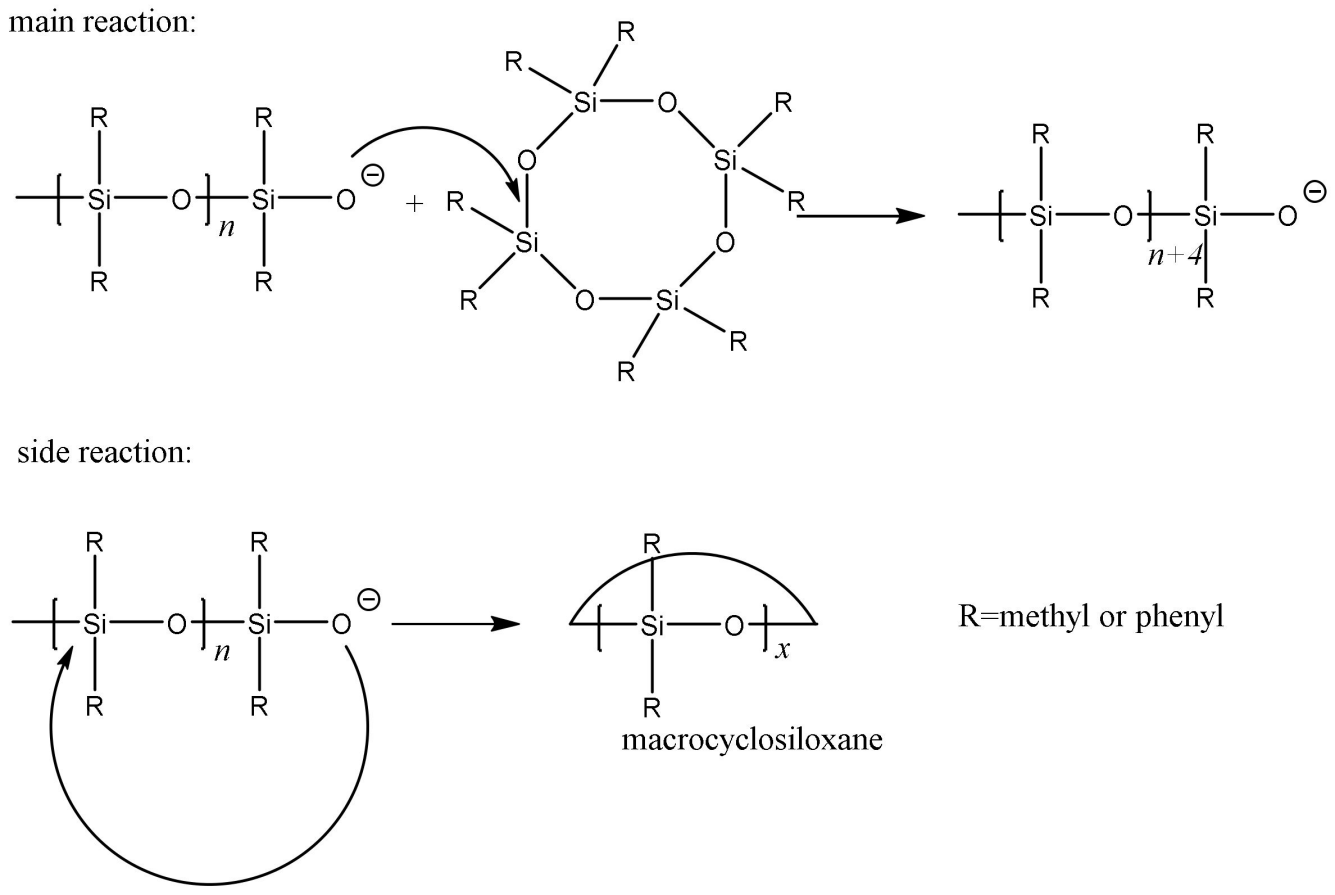
How the cyclosiloxane oligomer impurity of RTV phenyl silicone rubber is produced during the silicone rubber synthesis process.

As mentioned above, the pyrolysis process of RTV phenyl silicone rubber vulcanized by TEOS and TPOS could divided into three stages and the pyrolysis process of RTV phenyl silicone rubber vulcanized by polysilazane could only be divided into two stages. For the first pyrolysis stages of RTV phenyl silicone rubber vulcanized by TEOS, TPOS, and polysilazane, the weight loss after ablation in the muffle roaster were all about 10%, and there was almost no SiO_2_ or SiC produced. All of the data demonstrated that the first pyrolysis stage was cyclosiloxane impurities of the RTV phenyl silicone rubber vapor. The impurities were produced during the process of silicone rubber chain growth, since the Si–O bond energy of the RTV phenyl silicone rubber polymer chain was similar to the Si–O bond energy of cyclosiloxane monomers, and macrocyclopolysiloxane residue would be produced during the polymer chain growth, as shown in Figure 7. It should be noticed that TG-MS could not detect the signal of the macrocyclopolysiloxane impurities, resulting from the fact that the molecular range of TG-MS was only 0 to 400 Da.

**Figure 8:**
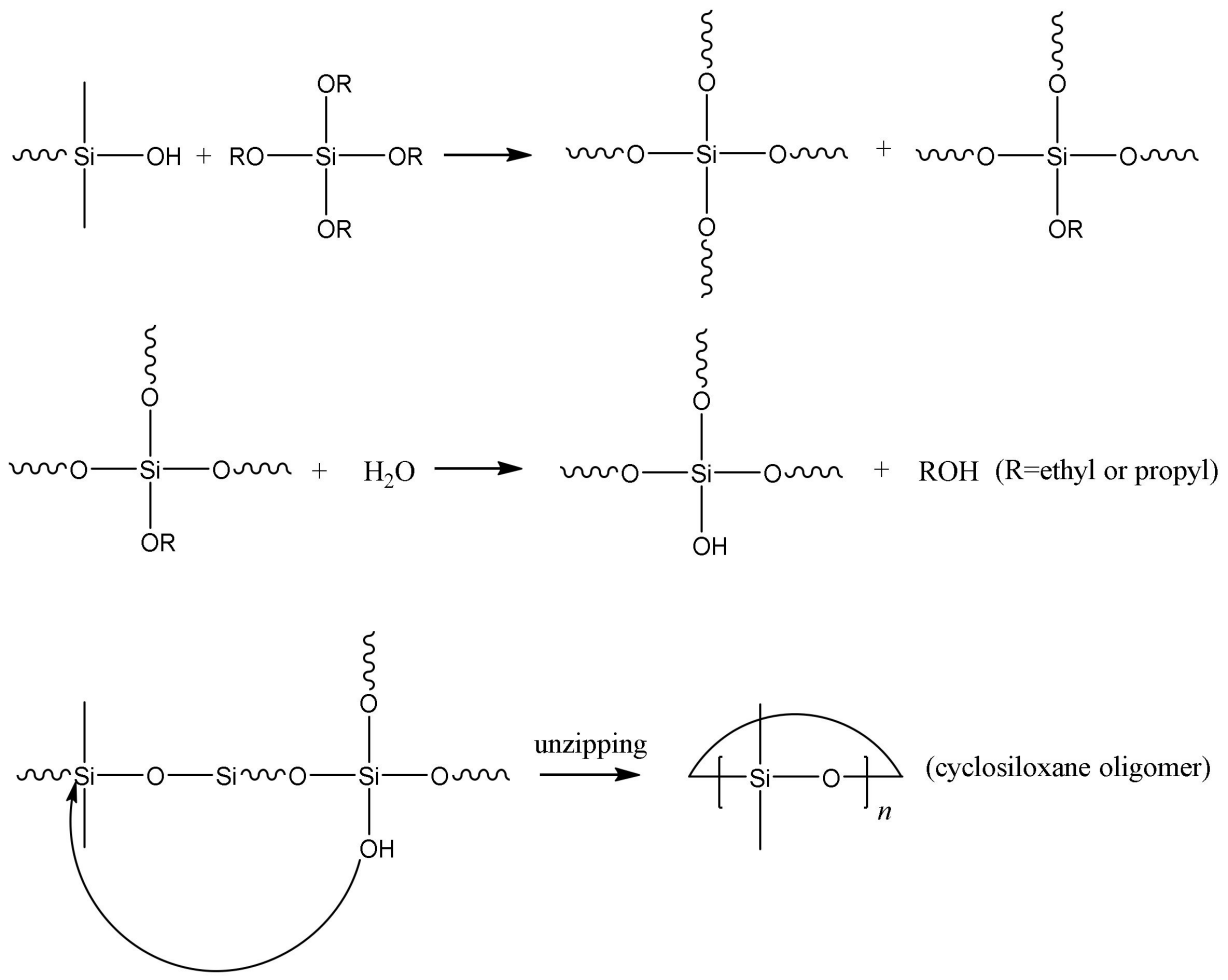
Unzipping mechanism of the second pyrolysis reaction stage of the RTV phenyl silicone rubber vulcanized by TEOS or TPOS.

For the second pyrolysis stages, the pyrolysis processes of RTV phenyl silicone rubber vulcanized by TEOS and TPOS were almost the same, but TPOS has shown better thermal stability and a slower degrade rate, due to the pyrolysis process through the mechanism of ‘unzipping’. As previous references report, the ‘unzipping’ pyrolysis reaction was initiated by the residue hydroxyl groups on the polysiloxane polymer chain. During the curing process of the RTV phenyl silicone rubber, several of the crosslinking agents could not react completely, and the ethoxy or oxypropyl groups might produce residues. The residual ethoxy or oxypropyl groups could hydrolyse and produce hydroxyl and initiate the ‘unzipping’ degradation reaction, as shown in Figure 8. Considering that the steric hindrance effect was stronger in propyl than in ethyl, the crosslinking density of RTV phenyl silicone rubber was lower when vulcanized by TPOS than by TEOS. Moreover, the hydrolysis was more difficult in oxypropyl than in ethoxy. Therefore, the residual hydroxy of RTV phenyl silicone rubber was less when vulcanized by TPOS than by TEOS, thereby leading to a more drastic pyrolysis reaction of the RTV phenyl silicone rubber vulcanized by TEOS than by TPOS at the second stage. Interestingly, silicone rubber vulcanized by polysilazane does not have this pyrolysis stage, resulting from the fact that the curing reaction of polysilazane has a higher conversion than TEOS and TPOS. Hence, hydroxyl would seldom produce residue and the “unzipping” degradation reaction would not react.

For the final stage, RTV phenyl silicone rubber vulcanized by polysilazane has poor thermal stability and a fast degradation ratio, due to the degradation reaction of the final pyrolysis stage that was reacted via the “random scission” mechanism. As the branching degree of polysilazane is lower than TEOS and TPOS, the crosslinking density of the RTV phenyl silicone rubber vulcanized by polysilazane was low, and the main polymer chain was flexible. The flexible polysiloxane chain could easily initiate the ‘random scission’ degradation.

## 4. Conclusions

In this work, the influence of crosslinking agents on the thermal stability of RTV phenyl silicone rubber was explored. The muffle roaster shows that RTV phenyl silicone rubber vulcanized by polysilazane has a better thermal stability than RTV phenyl silicone rubber vulcanized by TEOS and TPOS. After that, TG analysis showed that the RTV phenyl silicone rubber vulcanized by polysilazane has the optimum thermal stability, and the thermal pyrolysis reaction could be divided into two stages. The thermal pyrolysis reaction of the RTV phenyl silicone rubber vulcanized by TPOS and TEOS could be divided into three stages, and the RTV phenyl silicone rubber has a better thermal stability when vulcanized by TPOS than by TEOS at the second pyrolysis stage. Afterwards, the pyrolysis reaction mechanism was analyzed by TG-MS and pyGC-MS. In conclusion, the RTV phenyl silicone rubber vulcanized by polysilazane has the optimum thermal stability because the main polymer chain depolymerisation was only initiated by the ‘random scission’ process. The RTV phenyl silicone rubber has better thermal stability when vulcanized by TPOS than by TEOS at the second stage because it has less hydroxy group residue than TEOS. This work illustrated the pyrolysis process of the RTV phenyl silicone rubber vulcanized by TEOS, TPOS, and polysilazane, and provided a reference for studying the thermal pyrolysis process of silicone-based materials.

## Figures and Tables

**Table 1:** Element analysis of RTV phenyl silicone rubber after ablation.

Sample	Si (mol %)	O (mol %)	C (mol %)
TEOS–300 °C	26.45	26.04	47.51
TEOS–400 °C	35.03	29.75	35.22
TEOS–600 °C	37.50	40.13	22.37
TEOS–800 °C	41.01	50.66	8.33
TPOS–300 °C	25.85	25.33	48.82
TPOS–400 °C	33.03	29.10	37.87
TPOS–600 °C	36.00	40.04	23.96
TPOS–800 °C	41.00	50.50	8.50
Polysilazane–300 °C	26.45	26.04	47.51
Polysilazane–400 °C	29.34	29.75	40.91
Polysilazane–600 °C	33.88	35.66	30.46
Polysilazane–800 °C	41.01	50.66	15.56
